# Demographic and disease representativeness of Childhood Arthritis and Rheumatology Research Alliance (CARRA) Registry participants with lupus: a single center retrospective cohort

**DOI:** 10.1186/s12969-026-01187-7

**Published:** 2026-01-28

**Authors:** Rebecca L. Hetrick, Michaela Harter, Jennifer M. P. Woo, Martha Rodriguez, Amy Rakestraw, Stacey E. Tarvin

**Affiliations:** 1 Indiana University School of Medicine, Riley Hospital for Children at IU Health, 1120 West Michigan St. CL200, Indianapolis, IN 46202 USA; 2 Epidemiology Branch, National Institute of Environmental Health Sciences, Research Triangle Park, NC USA

**Keywords:** Systemic lupus erythematosus, Pediatric rheumatic disease, Health equity research, Representation of race and ethnicity, Research registries

## Correspondence

Equitable representation in medical research is necessary for generating high-quality evidence and improving health outcomes. The persistent underrepresentation of minoritized groups in medical research has been well documented across disciplines [1], including in studies of adult lupus patients [2, 3]. However, less is known about how these patterns may manifest in pediatric populations. This pilot study evaluated the demographic and clinical representativeness of patients with childhood-onset systemic lupus erythematosus (cSLE) enrolled in the Childhood Arthritis and Rheumatology Research Alliance (CARRA) Registry at a single urban tertiary care center by comparing them to the underlying population of cSLE patients.

We conducted a thorough chart review of all patients with cSLE (ICD-10: M32.x) who received care between January 2017 and September 2023 (*N* = 174). Demographic and clinical characteristics of CARRA Registry enrollees (*n* = 20) were compared to those of the site’s larger population using chi-square and Student’s t-test (Tables 1 and 2). Registry enrollees did not differ significantly from the broader site-based patient population in age, ethnicity, primary language, insurance status, neighborhood disadvantage, distance to care, and county level urbanization. However, Registry enrollees were significantly less likely to identify as non-White or multiracial (20% vs. 45%, X^2^ *p* = 0.03). No significant differences were observed in clinical characteristics, including disease activity, organ damage, time to diagnosis, intravenous steroids use at diagnosis, hospitalization at diagnosis, and renal involvement.

**Table 1:** Demographic and clinical characteristics of the Riley lupus cohort (*N* = 174)

Demographic Characteristics	
Age at diagnosis, median (IQR)	13 (11,15)
Sex, Female, n (%)	147 (85%)
Race, n (%)	
Asian Black or African American White Other Missing	14 (8%) 55 (33%) 93 (55%) 6 (4%) 6
Ethnicity, n (%)	
Hispanic or Latino Not Hispanic or Latino Missing	36 (21%) 137 (79%) 1
Primary Language, n (%)	
English Spanish Other Missing	151 (87%) 14 (8%) 8 (5%) 1
Payor Status, n (%)	
Medicaid or CHIP Private insurance TRICARE Uninsured	78 (45%) 91 (52%) 2 (1%) 3 (2%)
Indiana state resident, n (%)	168 (97%)
Area Deprivation Index State Decile, n (%)	
1–3 *(least disadvantaged)* 4–6 7–10 *(most disadvantaged)* Missing	49 (35%) 40 (28%) 53 (37%) 32*
Distance to care (miles), mean (SD)	68 (75)
County Level Urbanization	
Metropolitan Non-metropolitan	147 (85%) 27 (15%)
CARRA Registry status, n (%)	
Enrolled Not enrolled	20 (11%) 154 (89%)
**Clinical Characteristics**	
SLEDAI-2 K score**, mean (SD)	16 (9)
SLICC Damage Index, median (IQR)	0 (0,1)
Time to Diagnosis (weeks), n (%)	
<5 weeks 5–12 weeks >12 weeks Missing	42 (27%) 47 (30%) 67 (43%) 18
IV steroid pulse(s) within 3 months of diagnosis, n (%)	
No Yes Missing	104 (60%) 69 (40%) 1
Hospitalized for lupus-related conditions within 3 months of diagnosis, n (%)	
No Yes Missing	80 (46%) 93 (54%) 1
Renal involvement, n (%)	81 (47%)

*ADI calculated for Indiana resident with available 9-digit zip codes; **SLEDAI-2 K missing *n* = 37

Abbreviations: CHIP = Children’s Health Insurance Program; RUCC = United States Department of Agriculture Rural Urban Continuum Codes (2013); SLEDAI-2 K = Systemic Lupus Erythematosus Disease Activity Index 2000; SLICC = Systemic Lupus International Collaborating Clinics

**Table 2:** Comparison of demographic and clinical characteristics of Riley lupus cohort (*N* = 174) to registry enrollees (*N* = 20)

Demographic Characteristics	Total cohort (*N* = 174)	Registry enrollees (*N* = 20)	*P*-value^‡^
Age at diagnosis, median (range)	13 (15)	14 (9)	0.33^
Sex, n (%)			
Female	147 (85%)	18 (90%)	0.50
Race, n (%)			
Non-white or Multiracial White	75 (45%) 93 (55%)	4 (20%) 16 (80%)	0.03
Ethnicity, n (%)			
Hispanic or Latino Not Hispanic or Latino	36 (21%) 137 (79%)	7 (35%) 13 (65%)	0.12
Primary Language, n (%)			
English Non-English	151 (87%) 22 (13%)	19 (95%) 1 (5%)	0.30
Payor Status, n (%)			
Medicaid or CHIP Private insurance	78 (46%) 91 (54%)	9 (47%) 10 (53%)	0.92
Area Deprivation Index State Decile for Indiana Residents, n (%)			
1–3 *(least disadvantaged)* 4–6 7–10 *(most disadvantaged)*	49 (35%) 40 (28%) 53 (37%)	6 (32%) 5 (26%) 8 (42%)	0.91
County Level Urbanization, n (%)			
Metropolitan (RUCC 1–3) Non-metropolitan (RUCC 4–9)	147 (84%) 27 (16%)	16 (80%) 4 (20%)	0.58
Distance to Care, n (%)			
<60 miles *≥*60 miles	97 (56%) 77 (44%)	11 (55%) 9 (45%)	0.95
SLEDAI-2 K score, mean (SD)	16 (9)	19 (13)	0.30*
Presence of damage, n (%)			
No (SLICC Damage Index = 0) Yes (SLICC Damage Index > 0)	118 (68%) 56 (32%)	14 (70%) 6 (30%)	0.83
Time to Diagnosis (weeks), n (%)			
<5 weeks 5–12 weeks >12 weeks	42 (27%) 47 (30%) 67 (43%)	9 (45%) 6 (30%) 5 (25%)	0.14
Received IV steroid pulse(s) within 3 months of diagnosis, n (%)			
No Yes	104 (60%) 69 (40%)	8 (40%) 12 (60%)	0.07
Hospitalized for lupus-related conditions within 3 months of diagnosis, n (%)			
No Yes	80 (46%) 93 (54%)	6 (30%) 14 (70%)	0.15
Renal Involvement, n (%)			
No Yes	93 (53%) 81 (47%)	10 (50%) 10 (50%)	0.76

^**‡**^Chi-square unless otherwise specified; ^one-sample median test, *t-test

Abbreviations: CHIP = Children’s Health Insurance Program; RUCC = United States Department of Agriculture Rural Urban Continuum Codes (2013); SLEDAI-2 K = Systemic Lupus Erythematosus Disease Activity Index 2000; SLICC = Systemic Lupus International Collaborating Clinics

To date, no prior studies have assessed the representativeness of pediatric rheumatology research participants in North America. This pilot study offers regional insights into the demographic and clinical representativeness of CARRA Registry participants with cSLE. Overall, CARRA Registry enrollees at our site were demographically similar to the broader site cohort, with the exception of racial identity. Individuals identified as non-White or multiracial appeared underrepresented in the Registry. The limited sample size prevented further stratified analysis. Other limitations include unknown generalizability of data from a single site and the use of racial and ethnic data drawn from the electronic health record (EHR), as self-reported racial and ethnic identity remains the gold standard. Although our center has implemented a campaign to increase self-reported race and ethnicity collection, EHR-reported race and ethnicity can be error prone [4]. Reassuringly, among Registry enrollees at our site, agreement between self-reported and EHR-assigned race and ethnicity was strong (Cohen’s k = 0.83, 95%CI: 0.62-1.00, *p* < 0.0001).

In sum, this pilot study proves feasibility of the study methodology and underscores the need for larger, multi-site studies examining the representativeness of Registry data throughout the CARRA network.

## Data Availability

The datasets generated and/or analyzed during the current study are available from the corresponding author on reasonable request.

## Abbreviations

CARRA: Childhood Arthritis and Rheumatology Research Alliance
cSLE: Childhood-onset Systemic Lupus Erythematosus
EHR: Electronic Health Record
